# May Fillings Be Made Too Dense?

**Published:** 1871-06

**Authors:** W. E. Magill


### MAY FILLINGS BE MADE TOO DENSE?

BY W. E. MAGILL.

In the March number of "the DENTAL REGISTER," is an article entitled "Splitting Teeth with the Mallet." It seems now opportune to earnestly call the attention of intelligent members of our profession, to the facts narrated in that article, as they are but samples of an interesting class which is rapidly increasing under our modern modes of operating.

Nearly a year since, I asked the attention of our Lake Erie Association, to the fact that teeth filled with gold were sometimes broken asunder, or burst, or portions of the enamel thrown off, as if from internal force, and under such circumstances as to justify the conclusion that expansion of the metal produced the fracture. The observation of other members of the association coincided with my own. The inference drawn is this: that the use of adhesive gold enables us to fill a class of teeth formerly filled with amalgam, or allowed to remain unfilled; that the mallet packs into large cavities and frail teeth, much more closely and densely than can be, or usually is done, by hand pressure; that when the quantity of metal equals or exceeds the surrounding quantity of tooth structure, its greater ability to expand from heat, (or greater rapidity, which would produce the same effect,) results in breaking asunder the enclosing cylinder.

Reasons why this does not more frequently occur are found in the *shape* of *cavities*. If they take the form of a cone, with the base on the grinding surface, or if they flare from within outwards, where the greater body of metal is located, the expansion may be in such direction, or so governed, as not to do immediate or noticeable harm, but possibly showing itself in the form of displacement, at first slight, and charged to other causes. Again, imperfect packing of the gold, either at sides or center, may avert the danger, and a little difficulty in reaching certain points with the plugger, will thus prove the salvation of the tooth.

If, as we suppose, the *expansive* force of metal approximates its *contractile* power, it is immense. We know that if the smith does not make sufficient allowance for contraction, when the heated tire cools it will warp the felloes and spring the spokes of his wheel. We know that the contractile power of heated metal, is used to draw together the sides of heavy buildings, when dangerous fissures occur.

2d. Fillings may be so dense and unyielding, when inserted upon the grinding surface, or brought from the approximal to the grinding surface, in supplying the place of lost structure, in molars and bicuspids, that occlusion of the jaws will ultimately burst the tooth laterally, driving off one or both cusps. It is true that this may be prevented by persevering cutting until articulation is perfect; but every patient does not have a nice sense of touch, and the operator's eye can not always detect a line's difference in articulation. I sometimes approach the desired state by drawing slips of tissue paper between the teeth, continuing to cut the filling until those teeth which should touch, will hold the paper and prevent withdrawal.

Adhesive gold and a heavy mallet together, produce a dense inelastic plug. Such a plug, probably, must impinge with more force upon the tooth-wall, to make a tight joint, than a more elastic material. Perhaps we ought to make this question of elasticity a study. Under the old mode of filling with soft foil and hand pressure, we had usually an elastic filling, or at least, one less rigid and inelastic than those now made with adhesive gold; and yet it has been a common experience to find such fillings in the teeth, apparently perfect, at the end of ten or twelve years. Sometimes the more dense fillings, which we considered our best, if not thoroughly anchored by undercuts, would be found with a projected edge, and we felt satisfied that the motive power was in the metal, and there was no counter force to return it. I have seen fillings so soft that you could take them out with a pointed piece of wood, retained in the teeth for years, and when removing them have been surprised by the good condition of the tooth surface beneath.

These questions might be profitably discussed. On approximal or buccal surfaces, where no direct force is to be resisted, what degree of density is necessary to make a tight joint and protect against decay? This being the main object, is it in such cases of any advantage or disadvantage to go beyond such degree of density, if ascertained?

In view of the facts which have called out this discussion, would it not be well to fill frail teeth or very large cavities, partly with osteoplastic, or other material subject to less expansion and contraction than metal?

I confess to considerable interest in this subject, and to a tendency to be prolix, partly due to haste; but if able pens are hereby induced to do *justice* to the theme, mine has done its duty.

